# Efficient characterization of multiple binding sites of small molecule imaging ligands on amyloid-beta, tau and alpha-synuclein

**DOI:** 10.1007/s00259-024-06806-7

**Published:** 2024-07-02

**Authors:** Jens Sobek, Junhao Li, Benjamin F. Combes, Juan A. Gerez, Martin T. Henrich, Fanni F. Geibl, Peter R. Nilsson, Kuangyu Shi, Axel Rominger, Wolfgang H. Oertel, Roger M. Nitsch, Agneta Nordberg, Hans Ågren, Ruiqing Ni

**Affiliations:** 1 Functional Genomics Center, University of Zurich & ETH Zurich, Zürich, Switzerland; 2 Department of Physics and Astronomy, Uppsala University, Uppsala, Sweden; 3 Institute for Regenerative Medicine, University of Zurich, Wagistrasse 12, 8952 Zürich, Switzerland; 4 Laboratory of Physical Chemistry, Department of Chemistry and Applied Biosciences, ETH Zurich, Zürich, Switzerland; 5 Department of Psychiatry and Psychotherapy, Philipps-University Marburg, Marburg, Germany; 6 Department of Neurology, Philipps-University Marburg, Marburg, Germany; 7 Divison of Chemistry, Department of Physics, Chemistry and Biology, Linköping University, Linköping, Sweden; 8 Department of Nuclear Medicine, Inselspital, Bern University Hospital, University of Bern, Bern, Switzerland; 9 Divison of Clinical Geriatrics, Center for Alzheimer Research, Department of Neurobiology, Care Sciences and Society, Karolinska Institutet, Stockholm, Sweden; 10 Institute for Biomedical Engineering, University of Zurich & ETH Zurich, Zürich, Switzerland

**Keywords:** Alpha-synuclein, Amyloid-beta, Binding sites, In silico, Surface plasmon resonance, Tau

## Abstract

**Purpose:**

There is an unmet need for compounds to detect fibrillar forms of alpha-synuclein (αSyn) and 4-repeat tau, which are critical in many neurodegenerative diseases. Here, we aim to develop an efficient surface plasmon resonance (SPR)-based assay to facilitate the characterization of small molecules that can bind these fibrils.

**Methods:**

SPR measurements were conducted to characterize the binding properties of fluorescent ligands/compounds toward recombinant amyloid-beta (Aβ)_42_, K18-tau, full-length 2N4R-tau and αSyn fibrils. In silico modeling was performed to examine the binding pockets of ligands on αSyn fibrils. Immunofluorescence staining of postmortem brain tissue slices from Parkinson’s disease patients and mouse models was performed with fluorescence ligands and specific antibodies.

**Results:**

We optimized the protocol for the immobilization of Aβ_42_, K18-tau, full-length 2N4R-tau and αSyn fibrils in a controlled aggregation state on SPR-sensor chips and for assessing their binding to ligands. The SPR results from the analysis of binding kinetics suggested the presence of at least two binding sites for all fibrils, including luminescent conjugated oligothiophenes, benzothiazole derivatives, nonfluorescent methylene blue and lansoprazole. In silico modeling studies for αSyn (6H6B) revealed four binding sites with a preference for one site on the surface. Immunofluorescence staining validated the detection of pS129-αSyn positivity in the brains of Parkinson’s disease patients and αSyn preformed-fibril injected mice, 6E10-positive Aβ in arcAβ mice, and AT-8/AT-100-positivity in pR5 mice.

**Conclusion:**

SPR measurements of small molecules binding to Aβ_42_, K18/full-length 2N4R-tau and αSyn fibrils suggested the existence of multiple binding sites. This approach may provide efficient characterization of compounds for neurodegenerative disease-relevant proteinopathies.

**Supplementary Information:**

The online version contains supplementary material available at 10.1007/s00259-024-06806-7.

## Introduction

Neurodegenerative diseases represent a tremendous unmet clinical need. A common feature of these diseases is the abnormal cerebral accumulation and spreading of pathological protein aggregates, which affect selective vulnerable circuits in a disease-specific pattern [1]. Alzheimer’s disease (AD) is pathologically characterized by amyloid-β (Aβ) plaques and neurofibrillary tangles of hyperphosphorylated tau. Other tauopathies include frontotemporal dementia with 4R and 3R tau, progressive supranuclear palsy (PSP) with 4R tau, and corticobasal degeneration (CBD) with 3R tau accumulation [2]. α-Synucleinopathy is characterized by the accumulation of alpha-synuclein (αSyn) in Parkinson’s disease (PD), dementia with Lewy bodies and multiple system atrophy (MSA). The use of positron emission tomography (PET) with Aβ and tau imaging ligands has facilitated the early/differential diagnosis of AD [3].

Currently, there is an unmet clinical need for PET ligands for 4-repeat tau and αSyn aggregates to assist in diagnostic and clinical outcome evaluations. Several imaging ligands are currently in the pipeline, e.g., [^18^F]ACI-12589 [4], [^18^F]F0502B [5], [^11^C]MODAG-001 [6], [^18^F]SPAL-T-06 [7] and [^18^F]UCB-2897 (NCT05274568). Optical imaging ligands have been developed and applied in mechanistic and treatment studies using animal models recapitulating amyloidosis/tauopathy/α-synucleinopathy. Several imaging ligands, such as BTA-1, methoxy-X04, BF-158, PBB5, luminescent conjugated oligothiophenes (LCOs), BODIPY derivatives and fluorescently labeled antibodies, have been used for two-photon and diffuse optical imaging [8–15]. A number of experimental techniques, including fluorescence assays, radioligand competition assays [16–19], nuclear magnetic resonance spectroscopy of αSyn [20, 21], and cryogenic electron microscopy (cryo-EM) [22, 23], have been used to investigate interactions between ligands and Aβ peptides [24–26] and have demonstrated multiple ligand binding sites. In silico studies have also often been applied to study the interactions between ligands and proteinopathies/fibrils [27–31].

The surface plasmon resonance (SPR) assay is the method of choice for studying the kinetics of interactions for a wide range of molecular systems and has been widely used in pharmaceutical/biosensing/biomolecular research [32, 33]. SPR has been more commonly used for detecting Aβ and tau monomers in biological samples, such as blood or cerebrospinal fluid, as diagnostic biomarkers [34–37]. It has also been used to investigate Aβ elongation, aggregation dynamics [38, 39] and interactions with aggregation inhibitors [40–43]. A few studies have reported the SPR of αSyn fibrils [44–48], tau fibrils [49–51], and Aβ fibrils [51, 52].

A general problem of SPR measurements of small molecule ligand binding to immobilized fibrils is the large molar mass ratio (small molecule compound: < 1000 Da, fibrils > 1 MDa), which requires high surface densities to achieve sufficient signal intensity. This situation is partly improved by the large number of binding sites at the protein complexes leading to higher stochiometric binding ratios. In a typical SPR experiment, ligand solutions in the concentration range of approximately 1/10 of *K*_D_ and 10 × *K*_D_ are injected into the flow cell system of the instrument. In the case of low-affinity (µM) binders, the injection of high nM or µM ligand solutions often leads to strong adsorption of positively charged and/or hydrophobic compounds, including the dyes investigated, to the negatively charged chip surface, which impairs data quality. To overcome problems with unspecific adsorption, a suitable chip surface had to be found for each combination of fibril and dye. Another problem is artifacts of unknown origin that disturb the sensorgram characteristics and, in some cases, render data evaluation impossible.

Here, we optimized SPR protocols to determine the binding kinetics of small molecules (imaging ligands and nonfluorescent compounds) on Aβ_42_, K18-tau, full-length 2N4R tau and αSyn fibrils. We further examined the binding sites of fluorescence ligands on αSyn fibrils by in silico modeling and immunofluorescence staining in postmortem human brain tissues from patients with PD and from preformed fibril (PFF)-injected mouse models.

## Materials and methods

### Chemicals and antibodies

Detailed information on the chemicals and antibodies used in the study was provided in STables 1 and 2. The excitation and emission spectra of the fluorescence-emitting compounds are summarized in STable 1.

### Recombinant Aβ_42_, K18-tau, full-length 2N4R tau and αSyn fibril production, characterization and detection by fluorescence ligands

Recombinant Aβ_42_, K18-tau, full-length 2N4R tau (441 aa) and αSyn were expressed [53, 54] and measured for binding with fluorescent ligands [55] as described previously (details in the supplementary material).

### Surface plasmon resonance assay

SPR measurements were conducted in HEPES-buffered saline (HBS, Teknova, USA) buffer at 20 °C using Biacore instruments T200 and S200 (Cytiva, Uppsala, Sweden). Aβ_42_, tau, and αSyn recombinant fibrils were immobilized in the flow cell of sensor chips produced by Xantec (Düsseldorf, Germany) and Cytiva by coupling fibril amine groups to sulfo-N-hydroxysuccinimide (sulfo-NHS)-activated carboxylic acid groups on the chip surface. Since no nonbinding fibril(s) were available, the reference flow cell remained empty. Different chip surfaces, including the carboxymethylated dextran surface CM5 (Cytiva, Sweden), the carboxymethyldextran hydrogel surface CMD200M, the linear polycarboxylate hydrogel surface HC30M and HC1500M, the linear polycarboxylate hydrogel (reduced charge) surface HLC30M, and the zwitterionic hydrogel surface ZC150D (all from Xantec, Germany), were tested to obtain a high immobilization density and low nonspecific adsorption of ligands. All steps for surface preparation and immobilization were conducted at a flow rate of 5 µL/min. The surface carboxylic acid groups were activated with 0.2 M sulfo-NHS and 0.1 M 1-ethyl-3-(3-dimethylaminopropyl)carbodiimide (EDC, Xantec) (CM5 and CMD200M: 0.05 M) in 5 mM 2-(N-morpholino)ethanesulfonic acid (MES buffer, abcr Swiss AG, Switzerland) at pH 5.0 for 550 s (CM5 and CMD200M: 180 s) using 5 mM MES (pH 6.5) as the running buffer. K18-tau and αSyn fibrils were diluted to 2.5–10 µM in 5 mM acetate buffer (Fisher Scientific, Switzerland) at pH 4.5 and coupled for 750 s at a flow rate of 5 µL/minute on all surfaces except for ZD150D. Surfaces were blocked with 1 M ethanolamine (Xantec, Germany) for 500 s. On the ZC150D chip, K18-tau, αSyn and Aβ_42_ were immobilized at 2.5–10 µM in 5 mM MES, pH 6.5, for 750 s to a surface activated with 0.5 M EDC and 0.1 M sulfo-NHS for 600 s in 5 mM MES, pH 6.5. Finally, the surface was blocked with 1 M glycine in 10 mM MES, pH 6.5.

Binding kinetics were measured after a stabilization period of a few hours, which was necessary due to a slight decrease in the baseline, presumably caused by the slow dissociation of the fibrils. The immobilized fibrils were stable for a few days. Kinetic constants for binding were determined by injection of a dilution series of 5–8 concentrations of ligand in HBS buffer at a flow rate of 30 µL/minute in duplicate ("full kinetics"). Since no regeneration conditions were found that did not lead to decomposition of the fibrils, single cycle kinetics [56] were measured in cases of slow dissociation by consecutive injections of 5 ligands followed by a dissociation period of 10 min.

The data was evaluated using BiaEvaluate v2.03 software (Cytiva, Sweden). Binding kinetics were analyzed globally using different kinetic models, including 1 + 1, a sum of two exponentials model ("heterogeneous ligand" in terms of BiaEvaluate, sum2exp), and a sum of three exponentials model (sum3exp) that was created using the script editor of the software. The software does not allow the creation of higher-order kinetic models.

### In silico modeling and calculations

αSyn fibrils were obtained from previous studies (PDB codes: 6H6B [57, 58] and 2N0A [29]). The protein preparation wizard module in the Schrödinger suite (V. 2021–3, Schrödinger LLC, New York, U.S.A.) was used to add hydrogen atoms and determine the protonation states of the ionizable residues. The initial structures of the tested compounds were drawn at the Maestro interface and optimized using the Ligprep module (Schrödinger Release 2021–3: LigPrep, Schrödinger, LLC, New York, U.S.A.). The Glide module was used for docking, in which we used normal inner and outer box sizes of 10 and 20 Å, respectively [59, 60]. The standard precision mode was used for dockings, while other settings were left as default in Glide. The top 1 scored binding pose of each ligand–αSyn complex was subjected to molecular dynamics (MD) simulations. All MD simulations were carried out for 100-ns production with the OPLS-4 force field [61] using the Desmond module. In each simulation, the protein‒ligand complex was centered into an orthorhombic box with a boundary buffer of 12 Å, and ~ 24,000 TIP3P water [62] molecules and counterions were added to neutralize the system. Additional Na^+^ and Cl^−^ were added to reach a 0.15 M salt concentration. Before the production simulations, the system was energy minimized and equilibrated with a Nose‒Hoover chain thermostat (300.0 K) [63] and Martyna-Bobias-Klein barostat (1.0 atm) [64] using the default protocol implemented in Desmond. The molecular mechanics (MM)/generalized born solvent accessibility (GBSA) [65] binding free energy for each ligand was averaged from a total of 200 snapshots evenly extracted from the 100-ns trajectories. The Prime module [Schrödinger Release 2021–3: Prime, Schrödinger, LLC, New York, 2021.] was used for the MM/GBSA calculations. The protein‒ligand complexes were refined and optimized using the OPLS4 force field with the variable dielectric surface generalized born (VSGB) continuum solvation model [66]. For the minimization, the residues within 5 Å of the ligand were included. After that, the MM/GBSA method implemented in the Prime module was used to rescore the binding poses. Root-mean-square deviation (RMSD) analysis of the average distance between a group of atoms (e.g., backbone atoms of the four binding sites on αSyn) was performed.

### Postmortem human brain tissue

Two PD patients, each with a clinical diagnosis confirmed by pathological examination of Lewy bodies (Braak LB 6, without tau and Aβ), were included in this study (detailed information in Table 1). Paraffin-embedded autopsied brain tissues from the medulla oblongata and cerebellum with high αSyn inclusion accumulation were obtained from the Netherlands Brain Bank (NBB), Netherlands (Table 2).

**Table 1:** Results of SPR measurements of αSyn fibrils and Aβ_42_ fibrils and K18 and full-length 2N4R tau fibrils

Fibril	Analyte	Surface	Surface density/ RU	*k*_a_/M^−1^ s^−1^	*k*_d_/s^−1^	*K*_D_/ M	RU (max)	χ^2^
αSyn	HS-169	CMD200M	2940	2.88E + 05	2.54E-03	8.8E-09	16.3	0.60
				1.45E + 05	4.18E-02	2.89E-7	45.4	
	HS-84	CMD200M	3400	1.161E + 04	5.45E-06	4.78E-10	22.5	0.59
				1.115E + 06	6.02E-02	5.40E-08	42.2	
	h-FTAA	CMD200M	3400	1.62E+05	2.86E-03	1.77E-08	2.8	0.05
				1.11E+05	5.82E-02	5.23E-07	19.3	
	q-FTAA	CM5	2940	9.07E + 05	1.52E-02	1.67E-07	26.7	0.99
				1.53E + 03	7.05E-04	4.61E-07	60.9	
	MB	CMD200M	3400	4.72E + 05	8.34E-01	1.77E-06	39.0	0.06
				4.57E + 02	1.71E-02	3.75E-05	107.0	
K18-tau	HS-84	CMD200M	8280	4.89E + 03	2.21E-03	4.52E-07	35.3	0.07
				3.59E + 06	6.33E + 0	1.76E-06	33.4	
	q-FTAA	CMD200M	8280	4.89E + 02	3.14E-03	6.419E-06	47.9	0.16
				2.798E + 03	5.85E-02	2.092E-05	32.1	
	h-FTAA	HC1500M	9010	5.32E + 01	1.49E-04	2.81E-06	5968	0.19
				2.70E + 02	3.02E-02	1.12E-04	900	
2N4R-tau	HS-169	ZC150D	2900	6.78E + 01	1.64E-05	2.41E-07	2216	2.23
				1.12E + 04	8.09E-03	7.23E-07	67.6	
	Lan	ZC150D	2900	1.15E + 02	1.74E-04	1.51E-06	2682	0.09
				5.03E + 03	4.26E-02	8.47E-06	93.6	
Aβ_42_	HS-169	ZC150D	1770	1.20E + 02	1.67E-05	1.39E-07	641	0.49
				5.79E + 02	1.05E-02	1.82E-05	367.3	
	Lan	ZC150D	1770	9.47E + 02	1.63E-04	1.72E-07	383	0.07
				3.49E + 03	3.81E-02	1.09E-05	98.9	

*k*_a_/M^−1^ s^−1^ rate constants of association; *k*_d_/s^−1^ rate constants of dissociation; *K*_D_/M equilibrium dissociation constant. The calculations were performed using a sum2exp kinetic model; *RU(max)* response units at steady state; *Lan* lansoprazole; *MB* methylene blue

**Table 2:** Information on brain tissue samples from patients with Parkinson's disease and animal models

Postmortem human brain sample										
No	Sex	Age (y)	PM delay (h)			Braak tau	Amyloid-β	Braak LB	Diagnosis	Region
1	M	61	7.5			1	O	6	PD	MO
2	M	65	4.8			0	O	6	PD	MO
Mouse brain sample										
Model						Number of mice	Sex	Age (m)	Region imaged	
αSyn PFF-injected mice						2	M	5	CeA, NAc, PPN, PAG	
arcAβ mice						2	M/F	18	Ctx	
pR5 mice						2	M/F	18	Hip	
Non-transgenic littermate mice						4	M/F	18	Ctx, Hip	

*Ctx* cortex; *CeA* central amygdala; *LB* Lewy body; *MO* medulla oblongata; *NAc* nucleus accumbens; *PD* Parkinson's disease; *PM* postmortem; *PPN* pedunculopontine nucleus; *PAG* periaqueductal gray; Two non-transgenic littermate mice for arcAβ and pR5 mice, respectively. For the αSyn PFF-injected mouse model, the injection of PFF in the brain was performed at 8 weeks of age, with the endpoint at 20 weeks of age (5 months)

### Animal models

Two transgenic arcAβ mice overexpressing the human APP695 transgene harboring the Swedish (K670N/M671L) and Arctic (E693G) mutations under the control of the prion protein promoter and two age-matched nontransgenic littermates of both sexes (18 months of age) [67, 68] (Table 2). Two transgenic MAPT P301L mice overexpressing human 2N/4R tau under the neuron-specific Thy1.2 promoter (pR5 line, C57B6. Dg background) and two age-matched nontransgenic littermates of both sexes (18 months of age) [69] (Table 2). For the αSyn PFF mouse model, two male C57BL/6 J mice (Charles River, Sulzfeld, Germany), 8 weeks old at the beginning of the experiment, were used (Table 2). Animals were housed in individually ventilated cages inside a temperature-controlled room under a 12-h dark/light cycle with ad libitum access to food and water. For induction of αSyn pathology, a total volume of 550 nL of αSyn PFFs (concentration 2.5 µg/µL) was stereotactically injected into the pedunculopontine nucleus (PPN) or substantia nigra pars compacta (SNc) as previously described [70]. Twelve weeks postinjection, the mice were sacrificed, and tissue was prepared for immunohistochemical analysis. arcAβ, pR5 and PFF injected mice were perfused under ketamine/xylazine (75/10 mg/kg or 50/4.5 mg/kg body weight*, i.p.* bolus injection) with ice-cold 0.1 M PBS (pH 7.4) and 4% paraformaldehyde (PFA) in 0.1 M PBS (pH 7.4). After perfusion, the mice were decapitated, and the brains were quickly removed and fixed for 1 or 3 days in 4% PFA (pH 7.4) and 3 days in 30% sucrose solution. The brains were then frozen on dry ice and stored at -80 °C until sectioning.

### Ex vivo immunofluorescence and microscopy

The details on the antibodies and ligand concentrations are described in STable 1. 2. Human paraffin-embedded brain sections (3 µm) were deparaffinized and rehydrated prior to antigen retrieval step (citrate buffer pH 6.0) in a microwave for 20 min at 98 °C. The sections were then costained with anti-αSyn (phospho-S129, pS129) antibody plus fluorescent secondary antibody. Ligands were incubated after secondary antibody incubation for 30 min. Sections were counterstained using 4’,6-diamidino-2-phenylindole (DAPI). Mouse brains were embedded in tissue freezing media (OCT Compound, Tissue Tek, USA) and cut into 30–40 μm thick consecutive coronal sections using a cryostat microtome (CM3050 S, Leica, Germany). All sections spanning the complete rostrocaudal extent of the brain were kept in the correct order and stored at 4 °C in cryoprotect solution (1:1:3 volume ratio of ethylenglycol, glycerol and 0.1 M PBS) until further processing [71]. Anti-αSyn pS129 antibodies, anti-phospho-tau antibodies (AT-8, AT-100), or anti-Aβ_1-16_ antibody 6E10 plus fluorescent secondary antibody were used as previously described on brain slices from αSyn PFF-injected mice, arcAβ mice [72], and pR5 mice [55]. Ligands were incubated with secondary antibody for 30 min. Sections were counterstained with DAPI. The brain sections were imaged at 20 × magnification using an Axio Observer Z1 microscope (whole brain slide scanner) and at 63 × magnification using a Leica SP8 confocal microscope (Leica, Germany) for colocalization. It took approximately 1–1.5 h to acquire the tilted human brain slices by a slide scanner in our experiment (20 ×). The time varied depending on the size of the sample. Lambda scans were performed using a Leica SP8 confocal microscope for the emission spectrum of the ligands on the staining as previously described to validate the signal [55]. The images were analyzed using Qupath [73] and Fiji [74] (NIH, U.S.A.). Colocalization analysis of the confocal microscopy images (probe and antibody channels) was performed by using Fiji, and Manders' coefficients (M1, M2) were computed.

## Results

### In vitro fluorescence binding assays in recombinant fibrils

**Fig. 1:**
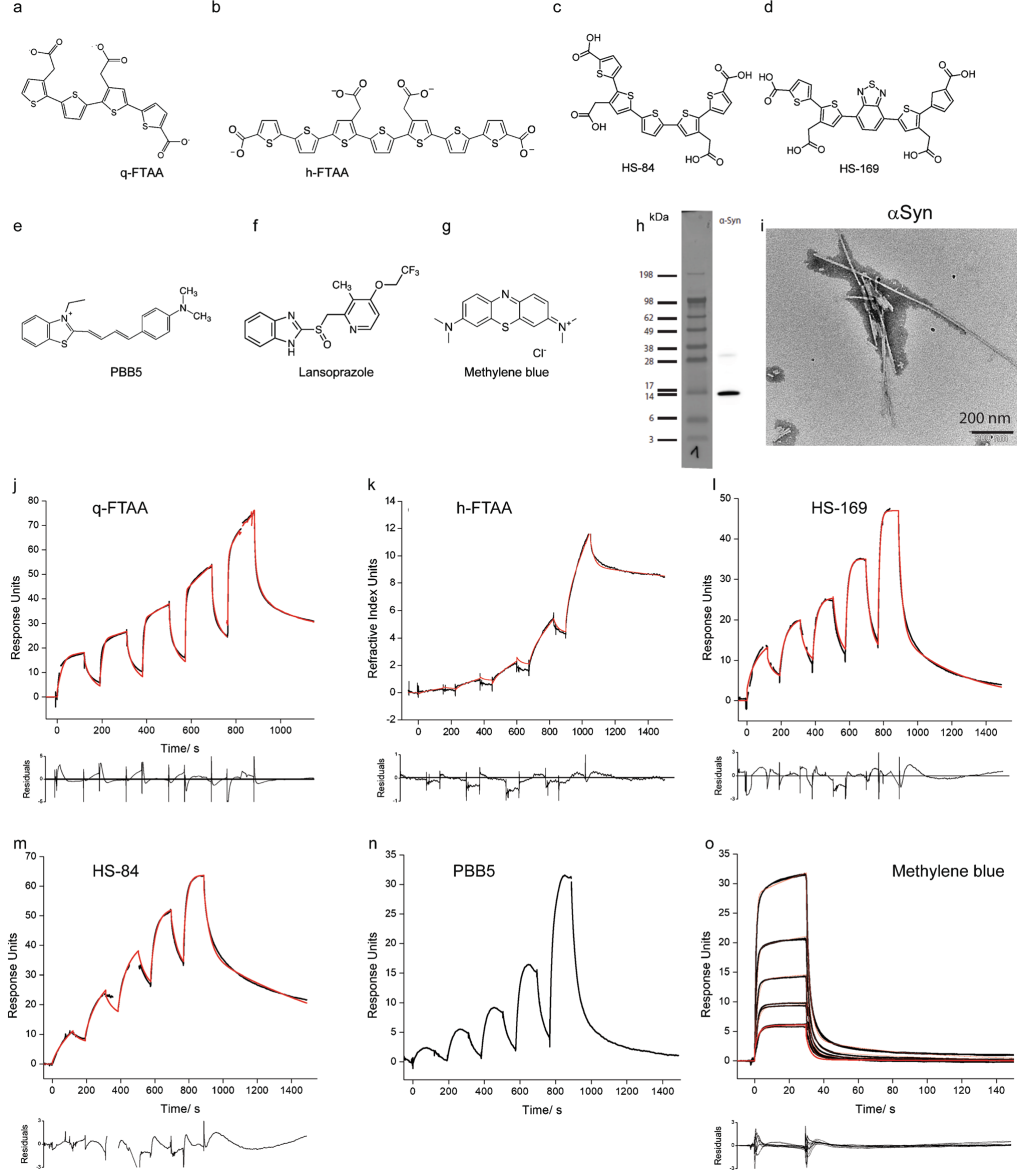
Characterization of ligand binding on recombinant alpha-synuclein fibrils. **a**-**g** Chemical structures of q-FTAA, h-FTAA, HS-84, HS-169, PBB5, lansoprazole and methylene blue. **h** Western blot of αSyn using anti-αSyn antibody, Syn211; **i** TEM of αSyn fibril; **j**-**o** Sensorgrams of q-FTAA(**j**), h-FTAA(**k**), HS-169(**o**), HS-84(**m**), PBB5(**n**), and methylene blue(**o**), binding to αSyn fibrils. The black line represents the experimental data, and the red line represents the fitted curve

**Fig. 2:**
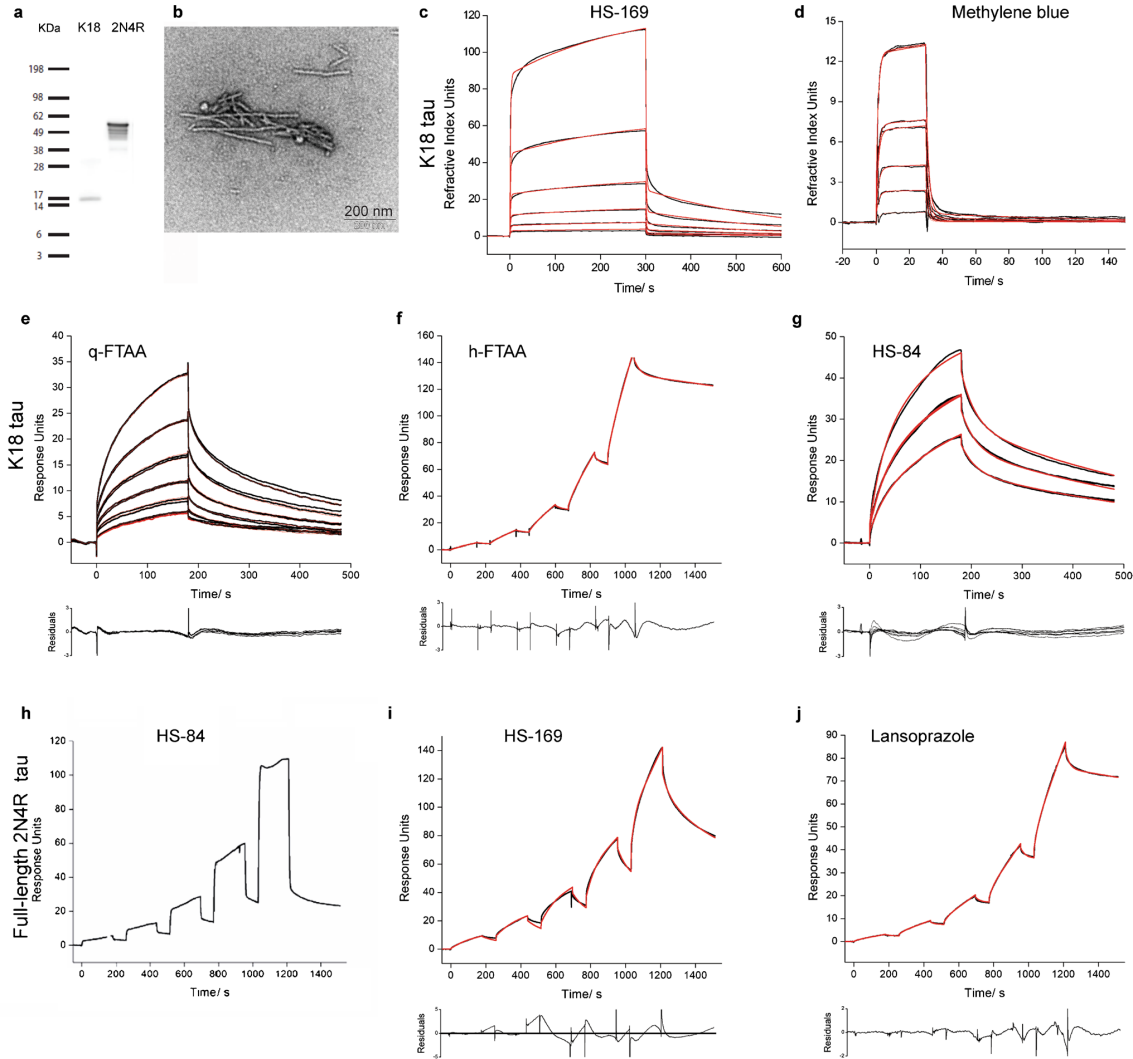
Characterization of ligand binding on recombinant K18-tau and full-length 2N4R tau fibrils. **a** Western blot of K18-tau and full-length 2N4R tau using anti-Tau(RD4) antibody, 1E1/A6; **b** TEM of K18-tau and full-length 2N4R tau fibrils; **c**-**g** Sensorgrams of HS-84(**c**), methylene blue(**d**), q-FTAA(**e**), h-FTAA(**f**), and HS-169(**g**) binding to K18-tau fibrils. (**h**-**j**) Sensorgrams of HS-84 (**h**), HS-169 (**i**), and lansoprazole (**j**) binding to full-length 2N4R tau fibrils. The black line represents the experimental data, and the red line represents the fitted curve

**Fig. 3:**
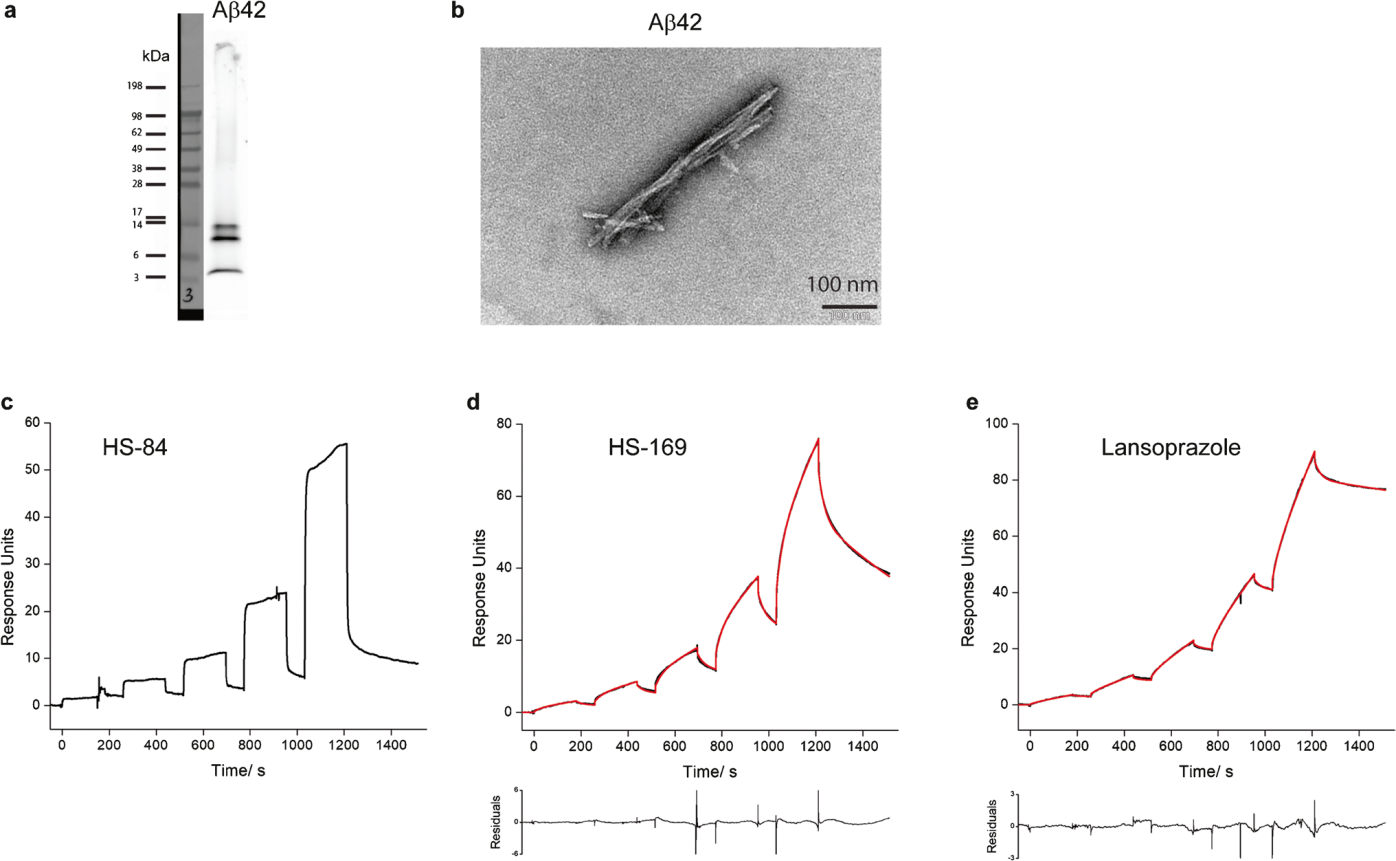
Characterization of ligand binding on recombinant Aβ_42_ fibrils. **a** Western blot of Aβ_42_ using the monoclonal anti-β-amyloid antibody BAM-10; **b** TEM image of Aβ_42_ fibrils; **c**-**e** Sensorgrams of HS-169, HS-84 and lansoprazole binding to Aβ_42_ fibrils. The black line represents the experimental data, and the red line represents the fitted curve

We produced Aβ_42_, K18-tau, full-length 2N4R tau and αSyn fibrils using bacterially produced recombinant monomers. During the processing of K18-tau, full-length 2N4R tau and αSyn after each step, an aliquot was taken, and at the end, identity was verified by SDS–PAGE (SFig. 1). The monomers were validated using western blotting, and the fibrils were validated using thioflavin T assays and TEM (Figs. 1, 2 and 3).

### SPR measurements of ligand binding to fibrils

The detailed protocol for the SPR measurements was described in the supplementary results section. Surfaces were tested to obtain sufficient immobilization of different fibrils and low nonspecific adsorption of the ligands/drugs. The best surface was chosen for different fibrils in the kinetic evaluation experiments, with the results shown in Table 1; In most cases, only one surface fulfilled the requirements for a valid measurement. For almost all ligands, the analysis of sensorgrams with a 1 + 1 kinetic model revealed poor fits due to high values of the least square error (χ^2^) and a nonrandom distribution of residuals. Kinetic models of higher order, including the sum2exp and sum3exp models, are better suited, which leads to good fits (Figs. 1, 2 and 3, Table 1). Kinetic data obtained with the sum2exp model revealed that in most cases, binding occurs in the high-nM to low-µM range. Only a few interactions of αSyn fibrils with HS-84 and the minor components of HS-169 and h-FTAA have affinities in the lower nM range.

Diffusion-controlled reactions of small molecules have rate constants on the order of 10^9^ M^−1^ s^−1^. However, from experience with the kinetics of small molecule binding to proteins, fast association constants are in the range of 10^5^–10^6^ M^−1^ s^−1^ and above. Values as low as 10^3^ M^−1^ s^−1^ and lower are considered slow. We found that some ligands bind to fibrils very slowly, as indicated by small association rate constants lower than 10^3^ M^−1^ s^−1^. This was observed for the interaction of q-FTAA with αSyn and K18-tau fibrils (Figs. 1 and 2); h-FTAA with K18-tau fibrils (Fig. 2); and for HS-169 and lansoprazole with both full-length 2N4R tau and Aβ_42_ fibrils (Figs. 2 and 3), respectively. Furthermore, we observed a number of artifacts of unknown origin impairing data quality, including large jumps (not shown) and a decrease in the signal during injection for αSyn/PBB5 (Fig. 1n).

It is noted that there is a difference between full kinetics and single-cycle (sc) kinetics measurements. Full kinetics measurements are preferred since they have the advantage of a larger number of full injection-dissociation cycles that can be conducted resulting in more data for fitting. Sc kinetics are usually applied when dissociation is slow for the analyte to be dissociated completely (back to baseline) since only then can a new (full) cycle be started without compromising the following measurement. In Biacore instruments, sc kinetics are limited to 5 injections and a single dissociation phase.

### In silico modeling demonstrated multiple binding sites for LCOs on αSyn fibrils

**Fig. 4:**
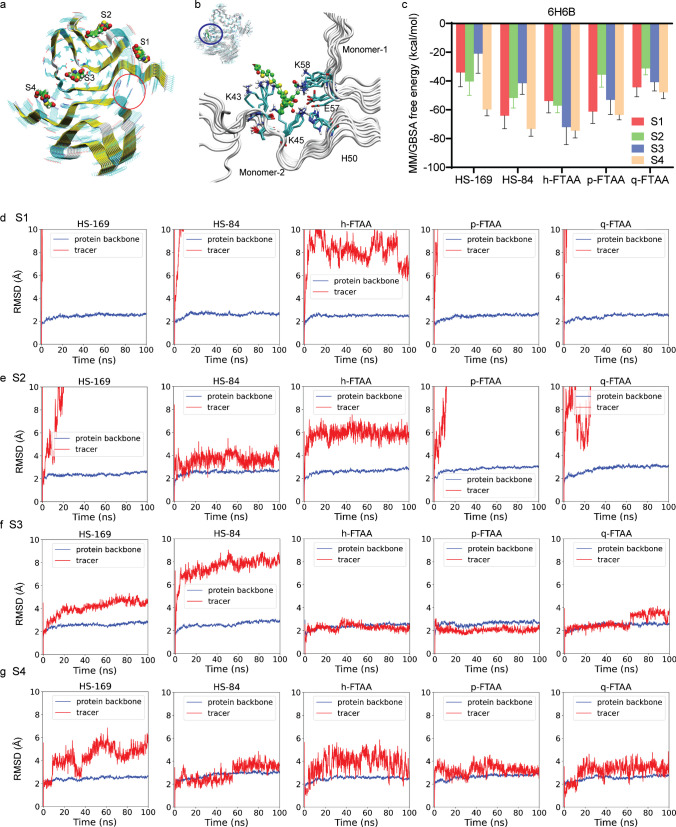
In silico modeling of the binding sites of HS-169, HS-84, h-FTAA, p-FTAA and q-FTAA on the 6H6B αSyn structure. **a** Four binding sites (S1-S4) on alpha-synuclein fibrils; the red circle indicates the location of site 4; **b** Zoomed-in view of h-FTAA binding to site 4. **c** MM/GBSA calculation of free energy indicating that site 4 is preferred by HS-169, HS-84, h-FTAA, p-FTAA and q-FTAA. **d**-**g** RMSD analysis of the ligands binding to 4 binding sites (S1-S4)

Here, we used the 6H6B structure from the recombinant αSyn fibril, which is assembled in paired helical fibril form. From the molecular docking studies of HS-169, HS-84, p-FTAA, and q-FTAA, we identified four binding sites on the αSyn fibril (6H6B, Fig. 4a, b), denoted sites 1–4. The salt bridge between E57 and H50 and the van der Waals interactions between the shallow hydrophobic residues (G51, A53, and V55) stabilize the paired fibrils in 6H6B. Site 3 is a core site inside the fibril and can be easily accessed when a limited number of molecules are used in the modeling, which might not be easily accessible in real situations. We carried out a 100-ns MD simulation for the binding of each ligand at each site, resulting in 20 trajectories with a total length of 2 μs. The ligands HS-169, HS-84, p-FTAA and q-FTAA are negatively charged; therefore, the binding affinities at site 4, which is a positively charged site, are more favorable than those at site 3 (Fig. 4c). In line with the docking studies, the ranking of binding free energies indicates that HS-169 and HS-84 strongly bind to site 4, followed by p-FTAA, q-FTAA and h-FTAA. These compounds are rich in hydrogen acceptors/donors and form stable hydrogen bonds with a cluster of positively charged residues K43, K45, H50, and K58 (Fig. 4c). The ligand RMSD to the initial conformation, usually used to estimate the stability of the binding, shows a deviation larger than 8 Å, which indicates that the ligand has moved away from the original binding site (Fig. 4d-g). From this point of view, the stability of the binding sites can be ranked as S4≈S3 > S2 > S1 for the binding of the five investigated ligands. We also note that the FTAA compounds were more stable in the core site 3 than in the dimer interface site 4. The positions of the acetate groups on the two neighbouring thiophene moieties of the centre thiophen appear to distinguish the stabilities of FTAAs (3′′*-yl* acetate) and HSs (4′′*-yl* acetate) in the interior core site 3 (SFig. 2). Additional in silico modelling was performed using the 2N0A αSyn structure. Four binding sites for DCVJ were demonstrated on the 2N0A αSyn structure [29]. Here, we found a preference of these ligands for core site 3 using the 2N0A αSyn structure (SFigs. 3).

### Validation using immunofluorescence staining of postmortem human brain tissue and mouse models

**Fig. 5:**
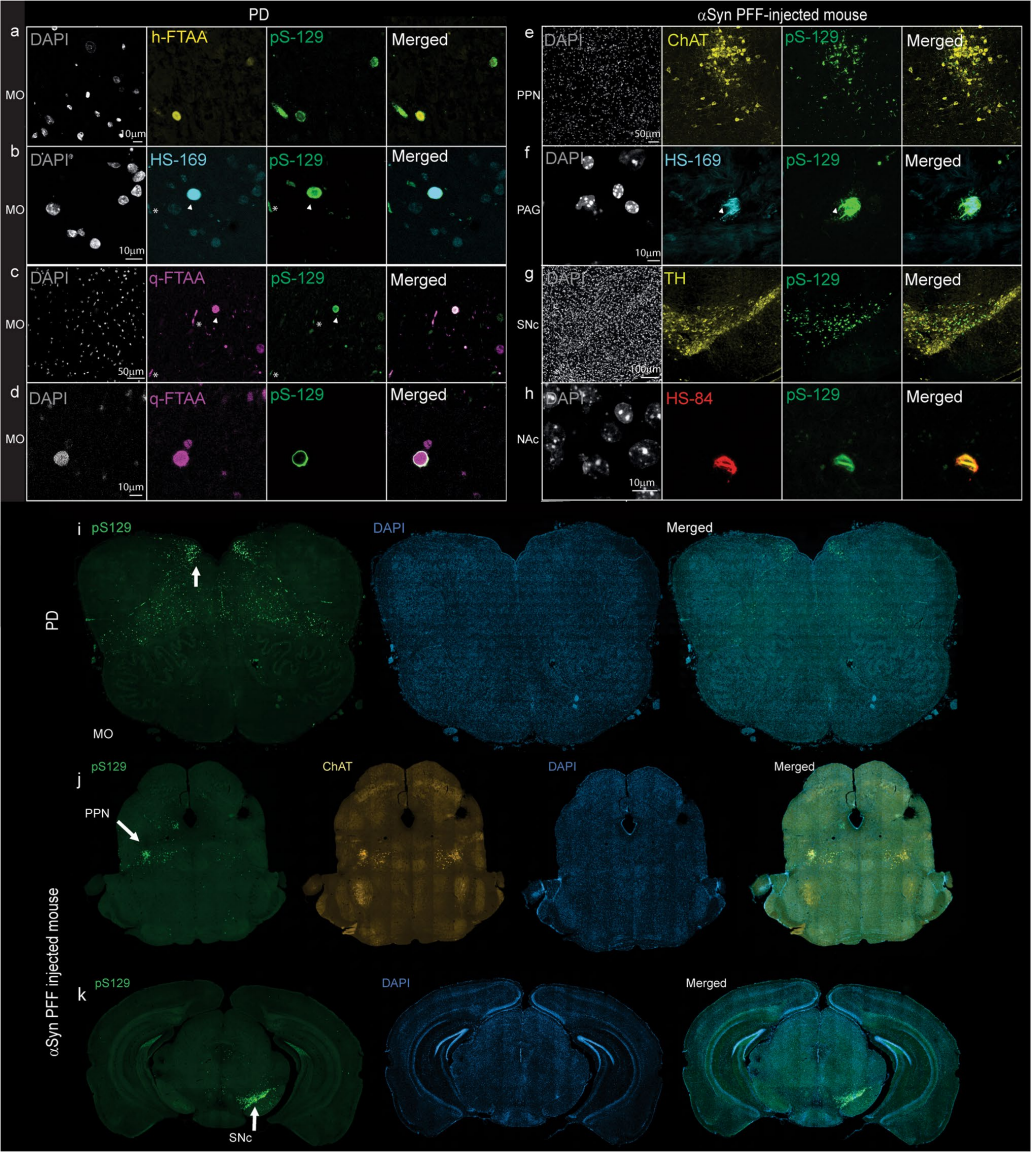
Confocal imaging of alpha-synuclein inclusions in brain tissue sections from PD patients and αSyn PFF-injected mice. **a**-**d** Colocalization of the Alexa488-anti-αSYN phosphor S129 antibody pS129 (green) with h-FTAA (yellow), HS-169 (cyan), and q-FTAA (magenta) in the medulla oblongata of PD patients (whole region image in i). αSyn-positive Lewy neurites (*) and Lewy bodies (arrowhead). **e**–**f**, **j** pS129-positive inclusions in ChAT-positive neurons (yellow) in the pedunculopontine nucleus (PPN) of αSyn-PFF-injected mice, with whole-brain images in j. (**f**) Colocalization of pS129-positive inclusions (green) with HS-169 (cyan) in the periaqueductal gray (PAG). **g**, **h** pS129-positive inclusions in TH-positive neurons (yellow) in the substantia nigra pars compacta (SNc) of αSyn-PFF-injected mice with whole-brain images in k). (**h**) Colocalization of pS129-positive inclusions (green) with HS-84 (red) in the nucleus accumbens (NAc)

We investigated the ligand detection of αSyn inclusions using an antibody that recognizes αSyn phosphorylated at serine 129 (pS129), as this epitope is a hallmark of Lewy bodies. We showed that ligands (h-FTAA, q-FTAA, and HS-169) were bound to pS129-positive αSyn inclusions in medulla oblongata tissue slices from two patients with PD (Figs. 5a-d, i). Colocalization of different ligands to both Lewy neurites and Lewy bodies was observed. Interestingly, the pS129 antibody (AB5336P) we used in the study appeared to detect the rim of the αSyn deposits, whereas q-FTAA detected the whole structure of the αSyn deposits (Figs. 5d). This difference is reflected by the relatively low coefficient of overlap between the q-FTAA and pS129 staining results (STable 3).

Next, we evaluated the effects of HS-169 and HS-84 on αSyn-PFF-injected mice at 12 weeks after injection into the PPN. A positive pS129 signal was detected in the ChAT-positive cholinergic PPN neurons of αSyn-PFF-injected mice (Fig. 5e, j). Colocalization of the ligands (HS-169 and HS-84) with the pS129 signal was observed in the PPN and periaqueductal gray of PPN-injected mice (Figs. 5e-g) and in the SNc as well as in the nucleus accumbens of the SNc-injected mice (Fig. 5h, k). The somatic pS129-positive αSyn aggregates appeared to be better stained by ligands than the neuritic pS129-positive αSyn aggregates (Fig. 5g).

**Fig. 6:**
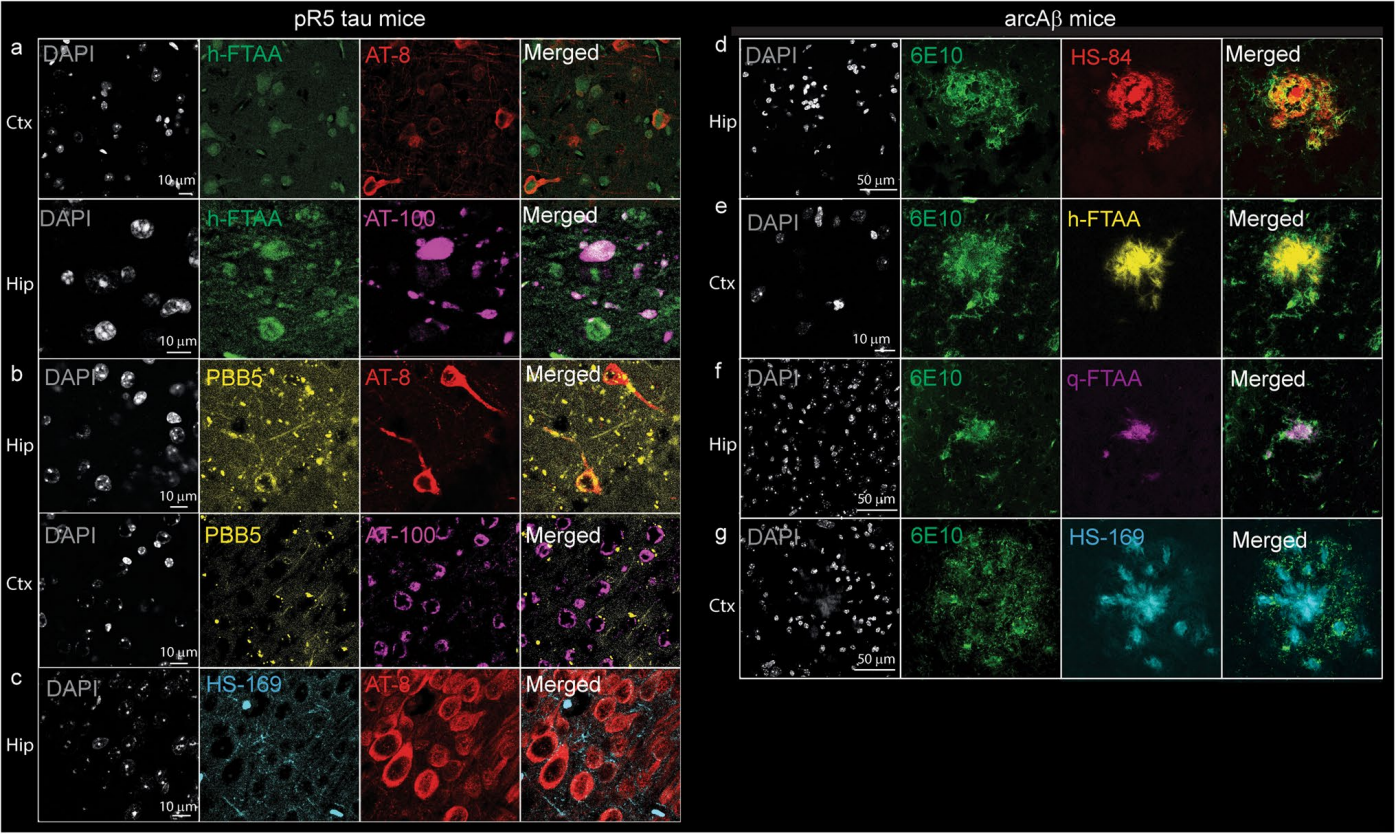
Confocal imaging of ligands with tau and amyloid-beta in mouse models. **a**-**c** Colocalization of tau staining by Alexa488-AT8 (red), Alexa488-AT100 (magenta) with h-FTAA (green), PBB5 (yellow), and HS-169 (cyan) in the hippocampus of pR5 tau mice; **d**-**g** colocalization of the anti-Aβ_1-16_ antibody Alexa488-6E10 (green) with HS-84 (red), h-FTAA (yellow), q-FTAA (magenta), and HS-169 (cyan) in the cortex of arcAβ mice. Nuclei were counterstained using DAPI (white)

To validate the detection of Aβ or tau pathology, we studied the binding of the aforementioned ligands to the brains of transgenic mice with Aβ or tau pathology. We chose transgenic mouse models to study Aβ or tau pathology since the brain tissue from AD patients commonly shows the coexistence of these two pathologies. Tau mainly accumulates in the cortex and hippocampus of pR5 mice. Immunofluorescence staining of coronal brain tissue sections from pR5 and nontransgenic mice costained with an anti-phospho-tau AT-8 antibody in the cortex and hippocampus confirmed the presence of PBB5 in tau inclusions (Fig. 6a-c). AT-100 antibody (mature phospho-tau) staining for PBB5 was performed on brain tissue slices from pR5 mice, and the results further validated the results (Fig. 6a, b).

We assessed ligand detection of Aβ deposits in brain tissue slices from arcAβ mice using 6E10. arcAβ mice develop Aβ pathology affecting both the brain parenchyma and vasculature from 6 months of age [67]. Immunofluorescence staining performed on coronal brain tissue sections from arcAβ and nontransgenic mice with ligands (h-FTAA, q-FTAA, and HS-169) costained with 6E10 (anti-Aβ_1−16_) antibody further confirmed the detection of parenchymal Aβ deposits in the cortical regions (Fig. 6d-g).

## Discussion

In the present work, SPR was used to characterize the binding of imaging ligands and small molecules to Aβ_42_, K18-tau, full-length 2N4R-tau and αSyn fibrils. The measured sensorgrams could not be evaluated by fitting a simple 1 + 1 kinetic model, suggesting that the binding kinetics are complex. Kinetic models of higher order, including the sum2exp and sum3exp models, were better suited for fitting the data. Based on the evident assumption of independent binding of a ligand to different sites, each interaction is characterized by a specific association and dissociation rate constant and its relative weight. In this case, the order of the fit model corresponds to the number of binding sites. Models of higher order, the sum2exp and, for some cases, the sum3exp model, considerably improved the fit results assessed by the least square error (χ^2^) and the distribution of residuals (Table 1, Figs. 1, 2 and 3).

However, it must be noted that fitting with the sum3exp model introduces considerable uncertainty to the kinetic constants due to the large number of initial parameters (*k*_a_, *k*_d_, and RU_max_ for each additional interaction, plus (optional) a parameter for the bulk response (RI) and a mass transfer coefficient), which can cause the fit routine to become stuck in local minima. This can be shown by repeating a fit with a single parameter being changed by an order of magnitude, a procedure that often does not reproduce the initial fit. Extensive tests with the sum3exp model revealed that the fit results show low resistance to changes in the initial parameters. For this reason, we limited the evaluation to the sum2exp model, although the residuals showed a more random distribution without a recognizable pattern, and χ^2^ was often much lower when the sum3exp model was applied and refrained from publishing fit results.

The number of exponentials needed to fit a sensorgram does not necessarily coincide with the number of binding sites identified in modeling calculations. In our interpretation, this represents the lower limit since some interactions may not be resolved due to the similarity of rate constants and the general limitation of SPR data quality. Therefore, we state that the results from the SPR measurements are in agreement with the modeling calculations and clearly support the notion of the existence of multiple binding sites in the fibrils even though their number cannot be precisely determined.

The binding of different ligands on the various sites of these fibrils with abnormal ligand binding cavities was also evaluated by free energy calculations to rank the affinities. In silico modeling suggested 4 binding sites on αSyn with a preference for certain binding pockets of different molecules (Fig. 4). which can be classified by the extent of solvent exposure. Sites S1 and S2 are similar due to the largest exposure to solvent, with similar dynamics for most of the docked ligands, of which the ligands left the original site quickly. Site S3 is the interior core site, which has the least solvent exposure, followed by site S4, which is located at the dimer interface. The binding of FTAA ligands in sites S3 and S4 were stable for the FTAA ligands during the MD simulations. Therefore, in view of the ligand fluctuations, molecular dynamics support the existence of similar binding sites as the binding sties from SPR study. However it is noted that SPR sensorgrams generally suffer from low temporal resolution (10–40 data points/s). For this reason, fitting with more than 2 exponentials does not yield reliable results. In contrast, in time-resolved fluorescence measurements by single-photon counting, millions of photons are typically collected, which allows 3 or even 4 exponentials to be fitted with high significance. This is not possible for SPR data analysis.

The stability of the binding sites identified by docking was further evaluated by MD simulations and binding free energy calculations. These studies provide atomic details and identify key amino residues for the interaction between ligands and fibrils. Here, an induced-fit mechanism of ligand binding, e.g., the enlargement of a narrow cleft in the protofibril when a large and planar ligand binds, is often observed. The ligand tends to increase its van der Waals or π-π interactions with the β-sheets of the protein, and due to a loss of molecular flexibility, the fluorescence quantum yield increases. The core site (site 3), which is buried inside the protofibril, can often exhibit high affinities for small molecules due to enhanced van der Waals interactions. However, here, we observed a surface site (S4) with strong binding affinities to the negatively charged ligands due to ionic interactions, indicating that the way the fibril twists is crucial for the existence of this site, which is surrounded by several base residues (K43, K45, H50, and K58). In addition to the binding free energies, the residence time of the ligands at the binding sites of the fibrils is also relevant to consider to rank the potency of the ligands, which can be estimated by the dissociation rate constant (*k*_d_) measured with SPR. If a ligand can preferentially bind to the core site(s) inside the protein fibrils, as in the present study, it is usually expected to have stronger binding affinity and longer wash-out time. Several recent studies have suggested the presence of 6 binding sites on Aβ fibrils [31, 75–79] and at least 4 binding sites on tau fibrils [28, 30, 80, 81]. Kuang et al. showed several surface and 3 core binding sites of PI-2620 [28] on tau fibrils and one core and 3 surface sites on αSyn fibrils [29].

Our affinity data for HS-169 and HS-84 are in line with available binding data for Aβ_42_ fibrils from previous studies. Using SPR, Johansson et al. immobilized azide-functionalized p-FTAA and showed a *K*_D_ of ~ 10 nM toward Aβ_42_, as did ratiometric comparison of the excitation spectra for free vs. bound dye [82]. Using competition studies with [^3^H]X-34, Bäck et al. showed that h-FTAA and q-FTAA bound to recombinant Aβ_42_ fibrils with EC_50_ values of ~ 250 nM and 330–630 nM, respectively [83]. In addition, Herrmann et al. performed a ratiometric comparison of the excitation spectra for free versus bound dye to determine the *K*_D_ values for some LCOs bound to recombinant PrP fibrils, and the *K*_D_ values were in the low nM range [84].

Autoradiography and in situ binding assays on brain tissues have also been used for evaluating binding affinity, but in general, only one binding site can be obtained [85]. For fluorescent probes, radiometric analysis has been used to estimate the binding sites with less accuracy. Using radioligand binding assays, multiple ligand binding sites on Aβ fibrils [17, 86, 87] and tau fibrils [16, 19, 88], e.g., THK-5351 [89], MK-6240 [90], and PBB3 [91], have been reported. Different tau ligands showed distinct binding towards tau in AD and different primary tauopathies [19, 22]. A recent cryo-EM density map revealed that a drug candidate bound to the core site of αSyn [23].

In addition to the affinity information, the rate constants for the different ligands were found to vary by orders of magnitude for the association, ranging from 10–10^6^ M^−1^ s^−1^ and 6 s^−1^ to 10^–5^ s^−1^ for the association and dissociation steps, respectively (Table 1). Exceptionally small association rate constants were found for some interactions, which are unusually slow, particularly for small molecules. We suggest that low association rate constant may be caused by the inaccessibility of ligands to the core sites, because there is usually a high barrier for ligand to be dissociated from a core site [29]. Therefore, the low affinity of the LCOs for the core site may also be a reason for the observed low association rate constant. There is no contradiction in the slow association and preference of S4. The reason is that a slow association rate constant combines with a slow dissociation rate constant, which results in a higher affinity compared to the other component. A similar slow association was observed by Sevenich et al. for a 16-mer peptide binding to aggregated αSyn [92].

The calculated equilibrium constants *K*_D_ are mainly in the nM-µM range, in agreement with the fact that these dyes have been successfully used in fluorescence imaging applications [8, 72]. Some targets show binding in the low nM range when dissociation is very slow, including αSyn, K18-tau fibrils with h-FTAA and Aβ_42_ fibril with lansoprazol. For the αSyn fibril, it is evident to assign the components found in the analysis of the sensorgrams to the binding sites calculated with molecular mechanics. In such a case, fast binding is expected to occur at sites that are easily accessible, e.g., S1 and S4 in Fig. 4. In contrast, slow association and dissociation components are attributed to core site 3, which is more difficult to access. For K18-tau and Aβ_42_ fibrils, no MM calculations are available. The similarity of the sensorgrams between αSyn suggests that multiple binding sites should also exist in these fibrils. In light of the difficulty in comparing rate constants calculated with higher-order kinetic models, a classification of targets based on the binding properties of the main component may be useful. Basically, there are two types of sensorgrams depending on whether the interaction is governed by a fast (Type 1) or slow (Type 2) association and dissociation (Table 1). In the case of slow dissociation and assuming that the interaction in vivo follows the same kinetics, the ligand is most likely suitable for imaging applications. As a consequence, screening by SPR can be useful for assessing whether a target is suitable for imaging applications. It must be noted that affinities determined at surfaces are typically an order of magnitude, or even more, lower compared with solution measurements.

In addition, similar to the theoretical calculations described here, other studies [84, 93] have shown that the interaction between positively charged lysine residues along the fibrils and the anionic carboxyl groups of the LCOs is crucial for binding. Fibrils are very large molecules and most likely have amine groups that are not part of a binding site. Since the fibrils are immobilized by the coupling of some of the fibril amine groups to sulfo-NHS-activated carboxylic acid groups on the chip surface, the binding mode of the LCOs might be distorted, as these lysine residues might not be accessible after immobilization. This can only be counteracted by a long linker (in the order of the molecular dimensions of the probe) that increases the distance to the surface or (partly by) a directed immobilization via specific reactive groups, which, as in our case, cannot be realized.

We demonstrated the colocalization of different fluorescence-emitting probes in brain tissue slices from PD patients, αSyn PFF-injected mice, and transgenic mice with Aβ plaques or tau inclusions, in line with previous observations [55, 68, 94]. The LCOs tested here appear to detect αSyn inclusions in the αSyn PFF-injected mouse brain and can be useful for in vivo imaging studies. Interestingly, we found that these beta-sheet binding chemical probes detected the entire structure of the αSyn deposits, while the pS129-αSyn antibody ab51253 detected mainly the rim of the structure (Fig. 5d), reflected by the low correlation coefficient. This has also been shown in previous studies on αSyn deposits in PD brain tissue slices using another LCO, HS-84 [95], as well as THK-565 (compounds of a different scaffold) [96]. This detection pattern is thus not specific to the q-FTAA. One possible reason is the better penetration of the chemical compounds to the aggregates. Moreover, an earlier study showed that C-terminal posttranslational modifications (PTMs) affect the detection of αSyn pS129 levels in primary neurons [97]. In comparison, chemical probes targeting a beta-sheet structure are less likely to affect the PTM of αSyn.

There are several limitations of our study. First, we did not use brain tissue-derived fibrils from patients with AD, primary tauopathy, PD, or MSA to investigate the ligand binding profiles. The binding sites on recombinant fibrils might differ from those on fibrils derived from patients [98]. Further studies using fibril pulldown from human brain tissue for potential strain-dependent affinity will provide important insight because LCOs have been shown to discriminate αSyn strains in PD and MSA [95, 99]. Second, we did not measure binding to oligomeric forms of Aβ, tau, or αSyn by SPR due to the uncertainty of the oligomer status on the chips as the fibril rapidly forms. With respect to the fitting of the SPR data, the sum3exp model used to adjust the number of binding sites found in the calculations resulted in low confidence, which was caused by the large number of free fitting parameters. Therefore, it was not reasonable to expect fitting with a sum of 4 exponential kinetic models to lead to useful results. Although most compounds investigated here are fluorescence ligand, we have included drugs such as lansoprazole and methylene blue in this study. The fluorescence ligand is easy to validate in the ex vivo fluorescence staining and colocalization analysis on the brain tissue. We will assess other PET imaging ligands/drug targeting A-beta/tau/alpha-synuclein in upcoming studies.

## Conclusion

In summary, we optimized the SPR assay and performed a systematic evaluation of SPR surfaces to characterize the binding of small molecules to Aβ_42_, K18-tau, full-length 2N4R tau and αSyn fibrils. Moreover, we provided calculation and modeling of the kinetic values of the ligands/drugs on the fibrils with sum3exp kinetic model. The application of this method can greatly improve the efficiency of screening and characterization of imaging ligands/drugs targeting these protein fibrils, which are critical for the diagnosis and treatment of neurodegenerative diseases such as AD, PD, and tauopathies. In addition, such a platform will also be potentially useful for studying competition of binding sites and off-target binding by displacement assays.

## Supplementary Information

Below is the link to the electronic supplementary material.

Supplementary file1 (DOCX 964 KB)

## Data Availability

The datasets generated and/or analyzed during the current study are available from the corresponding author upon reasonable request. The preprinted version of the manuscript has been deposited online at Biorvix [100].

## Abbreviations

*AD*: Alzheimer’s disease
*αSyn*: Alpha-synuclein
*Aβ*: Amyloid-beta
*CBD*: Corticobasal degeneration
*cryo-EM*: Cryogenic electron microscopy
*GBSA*: Generalized born solvent accessibility
*HBS*: HEPES-buffered saline
*hFTAA*: Hepta-formylthiophene acetic acid
*HS-84*: Name of a anionic pentameric oligothiophene
*HS-169*: Name of a pentameric D-A-D thiophene
*k*_d_: Dissociation rate constant
*LCO*: Luminescent conjugated oligothiophene
*MD*: Molecular dynamics
*MM*: Molecular mechanics
*MSA*: Multiple system atrophy
*PD*: Parkinson’s disease
*PET*: Positron emission tomography
*PPN*: Pedunculopontine nucleus
*PSP*: Progressive supranuclear palsy
*PFF*: Preformed fibrils
*RMSD*: Root-mean-square deviation
*qFTAA*: Quadro-formylthiophene acetic acid
*SPR*: Surface plasmon resonance
*SNc*: Substantia nigra pars compacta
*VSGB*: Variable dielectric surface generalized born
