# Supplementary material for: Efficient characterization of multiple binding sites of small molecule imaging ligands on amyloid-beta, tau and alpha-synuclein
Source: Eur J Nucl Med Mol Imaging. 2024 Jul 2;51(13):3960–77. doi: 10.1007/s00259-024-06806-7 (PMC11527973; doi:10.1007/s00259-024-06806-7)
Supplement: Supplementary file 1 — Supplementary file1 (DOCX 964 KB) [file 259_2024_6806_MOESM1_ESM.docx]

**Supplemental Methods**

Luminescent conjugated oligothiophenes (LCOs), including HS-84, HS-169, h-FTAA, and q-FTAA, were synthesized and provided by KPRN (Linköping, Sweden) [1, 2]. Methylene blue, lansoprazole (both Sigma Aldrich, Switzerland), and the (butadienyl) benzothiazole derivative PBB5 (RadiantDye, Germany) were purchased from the indicated sources (chemical structures in **Figs. 1a-g**). Detailed information on the chemicals and antibodies used in the study is provided in **STable 1, 2**.

**Recombinant Aβ_42_, K18 tau, full-length 2N4R** **tau and αSyn fibril production, characterization and detection by fluorescence ligands.**

Recombinant Aβ_42_, K18 tau, full-length 2N4R tau (441 aa) and αSyn were expressed and produced by *E. coli* as described previously [3, 4]. As a substrate for HRP, Pierce ECL and Western blotting substrate (Thermo Fisher Scientific, U.S.A.) were used to detect Aβ_42_ and αSyn. To detect K18 tau, ECL Prime Western blotting detection reagents (Cytiva, U.S.A.) were used. Images of the resulting blots were taken with an ImageQuant LAS 4000 (GE Healthcare). To induce fibrillization, the lyophilized proteins were dissolved in phosphate-buffered saline (PBS) buffer pH 7.4 (Gibco, U.S. containing 0.05% NaN_3_ (Sigma Aldrich, U.S.A.). The proteins were resuspended in 200 μl of PBS/NaN_3,_ and several aliquots were prepared in 1.5 ml Eppendorf tubes. For fibrilization, the protein solutions were incubated at 37 °C under agitation (500-700 RPM) in an Eppendorf thermomixer. The proteins were incubated at the following concentrations: Aβ_42_ (50 μM), αSyn (250 µM), and K18 tau (50 µM). The gel (Novex 10-20%, Tricine, Thermo Fisher Scientific, U.S.A.) was loaded with 10 μg of K18 tau and 10 μg of αSyn. For Aβ_42_, the quantity was 3.7 μg because of a higher dilution. The samples were mixed with Laemmli sample buffer and boiled at 95 °C for 5 minutes. Sodium dodecyl sulfate–polyacrylamide gel electrophoresis (SDS–PAGE) was performed at 100 V for 1 h 45 min. The gel was transferred to a nitrocellulose membrane (Invitrogen iBlot Transfer Stack, nitrocellulose, Thermo Fisher Scientific) using a dry blotting system (Invitrogen iBlot 2, Thermo Fisher Scientific, U.S.A.). The membrane was washed with 1 × PBS and 0.1% Tween 20 (PBST) and blocked with 5% fat-free milk. K18 tau was detected using a monoclonal tau antibody (anti-tau 4-repeat isoform RD4, clone 1E1/A6). Aβ_42_ was detected with a monoclonal Aβ antibody (clone BAM-10), and αSyn was detected using a monoclonal αSyn antibody (clone Syn211). The primary antibodies were diluted in 5% fat-free milk. As a secondary antibody, a purified anti-mouse IgG antibody conjugated to horseradish peroxidase (HRP) diluted in 5% fat-free milk was used (Jackson ImmunoResearch). The membranes were incubated with primary antibodies at 4 °C overnight. The membranes were washed with PBST, subsequently incubated with secondary antibodies at room temperature for 2 h and then washed with PBST. After incubation of the protein solutions, fibrillization was verified by thioflavin T fluorescence assay: 45 μL of thioflavin T (5 μM, Sigma Aldrich, U.S.A.) was mixed with either 2 μL of the incubated αSyn, 5 μL of K18 tau, full-length 2N4R tau or 5 μL of Aβ_42_ in a 45 µL quartz cuvette (quartz SUPRASIL Ultra-Micro Cell, Hellma GmbH, Germany).

**Transmission electron microscopy (TEM)**

TEM was performed by adding 4 μL of the fibril samples (~50 μM) in phosphate-buffered saline (PBS) directly to the negatively glow-discharged carbon-coated copper grids, followed by incubation for 1 minute at room temperature. The excess solution was gently removed using Whatman filter paper. Samples were stained with 10 μL of an aqueous phosphotungstic acid solution (1%, pH 7.2) for 1 minute. The excess stain on the grid was then wiped off with filter paper, washed with double-distilled water and air dried. Finally, the images were recorded on a Morgagni 268 electron microscope (FEI GmbH, Germany) at ScopeM, ETH Zurich.

**Supplemental results**

**Protocol optimization**

A general problem of SPR measurements of small molecule ligands binding to immobilized fibrils is the large molar mass ratio (small molecule compound: <1000 Da, fibrils >1 MDa), which requires high surface densities to receive sufficient signal intensity. The situation is partly improved by the fact that a large number of binding sites at the protein complexes lead to higher stochiometric binding ratios. Different chip surfaces were tested to achieve a high immobilization density and low unspecific ligand adsorption (see Methods, 2.3) (**Table 1, Figs. 1-3**).

In a typical SPR experiment, ligand solutions in the concentration range of approximately 1/10 of *K*_D_ and 10× *K*_D_ are injected into the flow cell system of the instrument. In the case of low-affinity binders, the injection of high nM or µM ligand solutions often leads to strong adsorption of positively charged and/or hydrophobic compounds to the negatively charged chip surface, which impairs data quality. This effect can be reduced by the use of a ZC150D surface, which is zwitterionic and slightly positively charged after NHS ester activation and fibril immobilization. Unspecific adsorption can amount to several hundred response units (RU), thus masking binding events or making reliable quantitation impossible. As a rule of thumb, the unspecific contribution to the signal should not exceed approximately 10% of the overall signal. In addition to the use of a suitable surface, this can be achieved by working at low ligand concentrations (below *K*_D_), which commonly results in a decrease in unspecific adsorption. For this reason, in the first test, ligands were injected for 30-60 s at a concentration of approximately 1 µM to the blank chip surface to determine their adsorption behavior.

**Supplementary Table** **1 List of compounds in the fluorescence assay, SPR assay**

| **Ligand** | **Conc [μM] in staining** | **Source** | **Excitation wavelength [nm]** | **Emission wavelength [nm]** | | | | |
| --- | --- | --- | --- | --- | --- | --- | --- | --- |
|  |  |  |  | Aβ_42_ | K18 tau | | αSYN | |
| PBB5 | 1.6 | RadiantDye, | 630 | 695 | | 688 | | 690 |
| HS-169 | 5 | KPRN | 375, 535 | 650 | | 640 | | 643 |
| HS-84 | 5 | KPRN | 430 | 504, 540 | | 504, 540 | | 504, 540 |
| h-FTAA | 5 | KPRN | 480 | 544, 573 | | 544, 577 | | 544, 577 |
| q-FTAA | 5 | KPRN | 430 | 473, 502, 533 | | 473, 502, 533 | | 473, 502 |
| Methylene blue |  | Sigma‒Aldrich | 680 | / | | / | | / |
| Lansoprazole |  | Sigma‒Aldrich | / | / | | / | | / |

MW, molecular weight. The second peak of h-FTAA and HS-84 differs between the binding to Aβ_42_, K18 tau and αSYN fibrils. The third peak (shoulder) of q-FTAA was more apparent in the binding to Aβ_42_ fibrils compared to K18 tau fibrils and was missing in the binding to αSYN fibrils.

**Supplementary Table 2 List of antibodies used for staining and for fibril characterization**

|  |  |  |  |  |
| --- | --- | --- | --- | --- |
| **Antibody/Compounds** | **Source** | **Cat. No.** |  | **Dilution** |
| DAPI | Sigma‒Aldrich | D9542-10MG |  | 1:1000 |
| Goat-anti-Rabbit Alexa488 | Invitrogen | A11034 | AB_2576217 | 1:200 |
| 6E10, Anti-β-amyloid, 1-16 antibody | Biolegend | 803001 | AB_2564653 | 1:1000 |
| AT-8, anti-Phospho-tau Ser202, Thr205 | Invitrogen | MN1020 | AB_223647 | 1:1000 |
| AT-100, anti-Phospho-tau Thr212, Ser214 | Invitrogen | MN1060 | AB_223652 | 1:1000 |
| Anti-α-synuclein pS129 (phospho-Ser129) | Abcam | ab51253 | AB_869973 | 1:1000 |
| Donkey anti-Rabbit Cy5 | Jackson ImmunoResearch | 711-175-152 | AB_2340607 | 1:200 |
| VECTASHIELD® Antifade | Vector Laboratories | H-1000-10 |  |  |
| Anti-Choline Acetyltransferase Antibody | Sigma‒Aldrich | AB144P-1ML5 | AB_2079751 | 1:100 |
| Tyrosine Hydroxylase Antibody | Merck Millipore | AB152 | AB_390204 | 1:1000 |
| Normal goat Serum (NGS) | Chemie Brunschwig | JAC005-000-121 |  |  |
| Normal Donkey Serum (NDS) | Interchim-Uptima | UP77719A K |  |  |
| Triton X-100 | Sigma‒Aldrich | X100-500ML |  |  |
| Anti-Tau (RD4) Antibody, clone 1E1/A6 | Sigma‒Aldrich | 05-804 | AB_11211556 | 1:1000 |
| Anti-α-Synuclein antibody, Mouse monoclonal, clone Syn211 | Sigma‒Aldrich | S5566 | AB_261518 | 1:2000 |
| Monoclonal Anti-β-amyloid antibody, clone BAM-10 | Sigma‒Aldrich | A5213 | AB_476742 | 1:1000 |
| Anti-mouse IgG antibody conjugated to horseradish peroxidase (HRP) | Jackson ImmunoResearch | 115-035-166 | AB_2338511 | 1:5000 |
| ECL Prime Western Blotting Detection Reagents | Cytiva | RPN2232 |  |  |
| Donkey-anti-Rat Alexa488 | Jackson ImmunoResearch | 712-546-153 | AB_2340686 | 1:400 |
| Goat-anti-mouse Alexa488 | ThermoFischer Scientific | A11001 | AB_2534069 | 1:200 |

**Supplementary Table 3 Analysis of overlapping between antibody and ligand in the staining in Figs. 5, 6**

| **Figure** | **Sample** | **Antibody (A)** | **Ligand (B)** | **M1, fraction of A overlapping B** | **M2, fraction of B overlapping A** |
| --- | --- | --- | --- | --- | --- |
| Fig.5a | PD | pS129 | h-FTAA | 0.404 | 0.4 |
| Fig.5b | PD | pS129 | HS-169 | 0.81 | 0.866 |
| Fig.5c | PD | pS129 | q-FTAA | 0.483 | 0.472 |
| Fig.5d | αSyn PFF injected mouse | pS129 | q-FTAA | 0.782 | 0.222 |
| Fig.5f | αSyn PFF injected mouse | pS129 | HS-169 | 0.673 | 0.84 |
| Fig.5h | αSyn PFF injected mouse | pS129 | HS-84 | 0.839 | 0.838 |
| Fig.6a | pR5 mouse | AT-8 | h-FTAA | 0.215 | 0.07 |
| Fig.6a | pR5 mouse | AT-100 | h-FTAA | 0.459 | 0.16 |
| Fig.6b | pR5 mouse | AT-8 | PBB5 | 0.39 | 0.09 |
| Fig.6b | pR5 mouse | AT-100 | PBB5 | 0.024 | 0.018 |
| Fig.6c | pR5 mouse | AT-8 | HS-169 | 0.164 | 0.279 |
| Fig.6d | arcAβ mouse | 6E10 | HS-84 | 0.607 | 0.6 |
| Fig.6e | arcAβ mouse | 6E10 | h-FTAA | 0.553 | 0.425 |
| Fig.6f | arcAβ mouse | 6E10 | q-FTAA | 0.447 | 0.588 |
| Fig.6g | arcAβ mouse | 6E10 | HS-169 | 0.658 | 0.462 |
|  |  |  |  |  |  |

PD: Parkinson’s disease; PFF: preformed fibril

**Supplemental Figures**


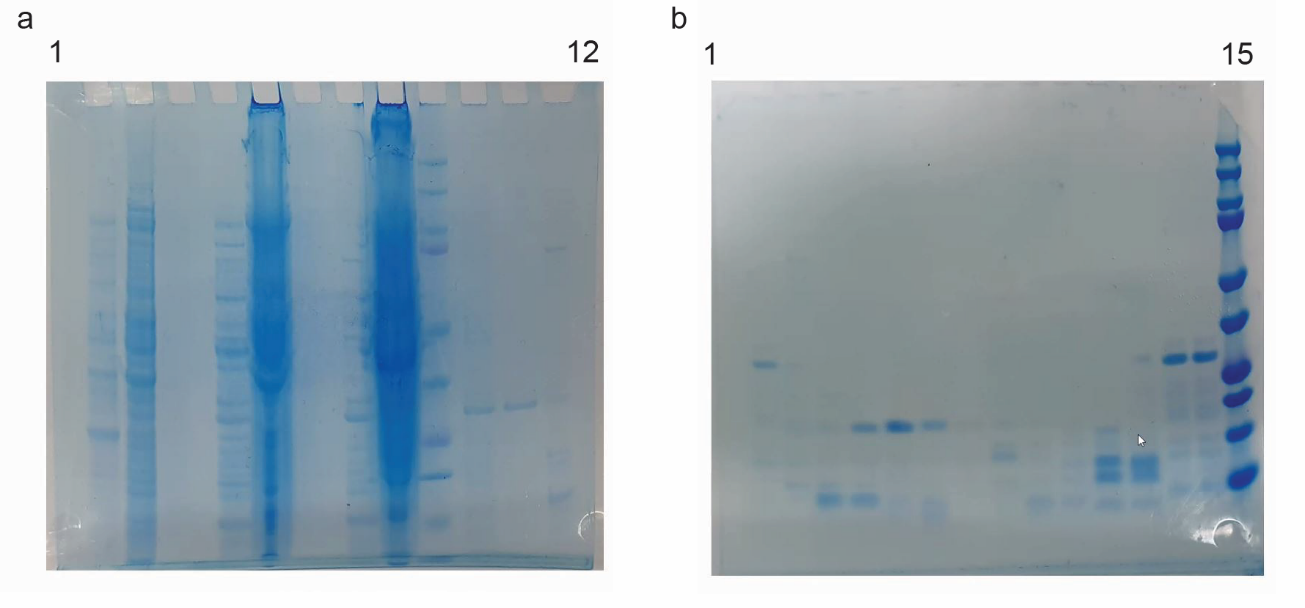


**Supplementary Fig 1. Confirmation of αSYN and K18 tau** (**a**) αSYN purification (bands 1-12). After each step, an aliquot was taken, and ultimately, the identity was verified by sodium dodecyl sulfate–polyacrylamide gel electrophoresis (SDS–PAGE). The identity of αSYN was confirmed by a visible band at approximately 14 kDa (in band 12). From bands 1-12 (left to right): 1) *E. coli* uninduced (before adding IPTG), 2) *E. coli* induced, 3) empty, 4) osmotic shock (OS) supernatant (Sup. ), 5) OS pellet, 6) empty, 7) heat shock (HS) supernatant, 8) HS pellet, 9) Bio-Rad Precision Plus Protein Dual Color Standards, 10) ammonium sulfate (AS) precipitation 35% supernatant, 11) AS 55% supernatant, 12) AS 55% pellet*.* (**b**) Tau purification (bands 1-15). After centrifugation of the cell debris, the supernatant was recovered, and column chromatography with a phosphocellulose column was performed. Twelve elution fractions of the chromatography were obtained and verified by SDS–PAGE (bands 1-12, left to right). The three fractions with the highest Tau concentrations (fractions 4, 5, and 6) were selected for subsequent dialysis and lyophilization. Bands 13-15 were the solution before chromatography, the flow-through, and the reference band (Bio-Rad Precision Plus Protein Dual Color Standards).


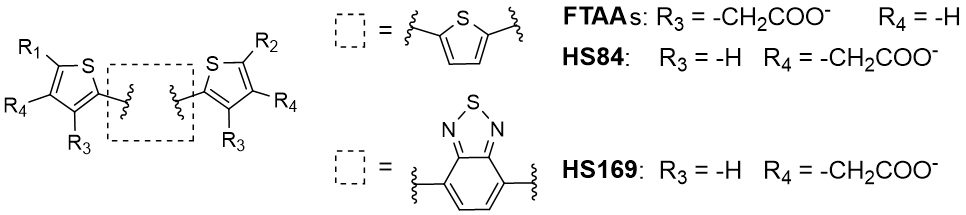


**Supplementary Fig. 2**. Location of the acetate substitution on the symmetrical thiophene moieties. For FTAAs, the acetate located at the 3′′ position seems to be more stable at core site 3 because of the fluctuations.


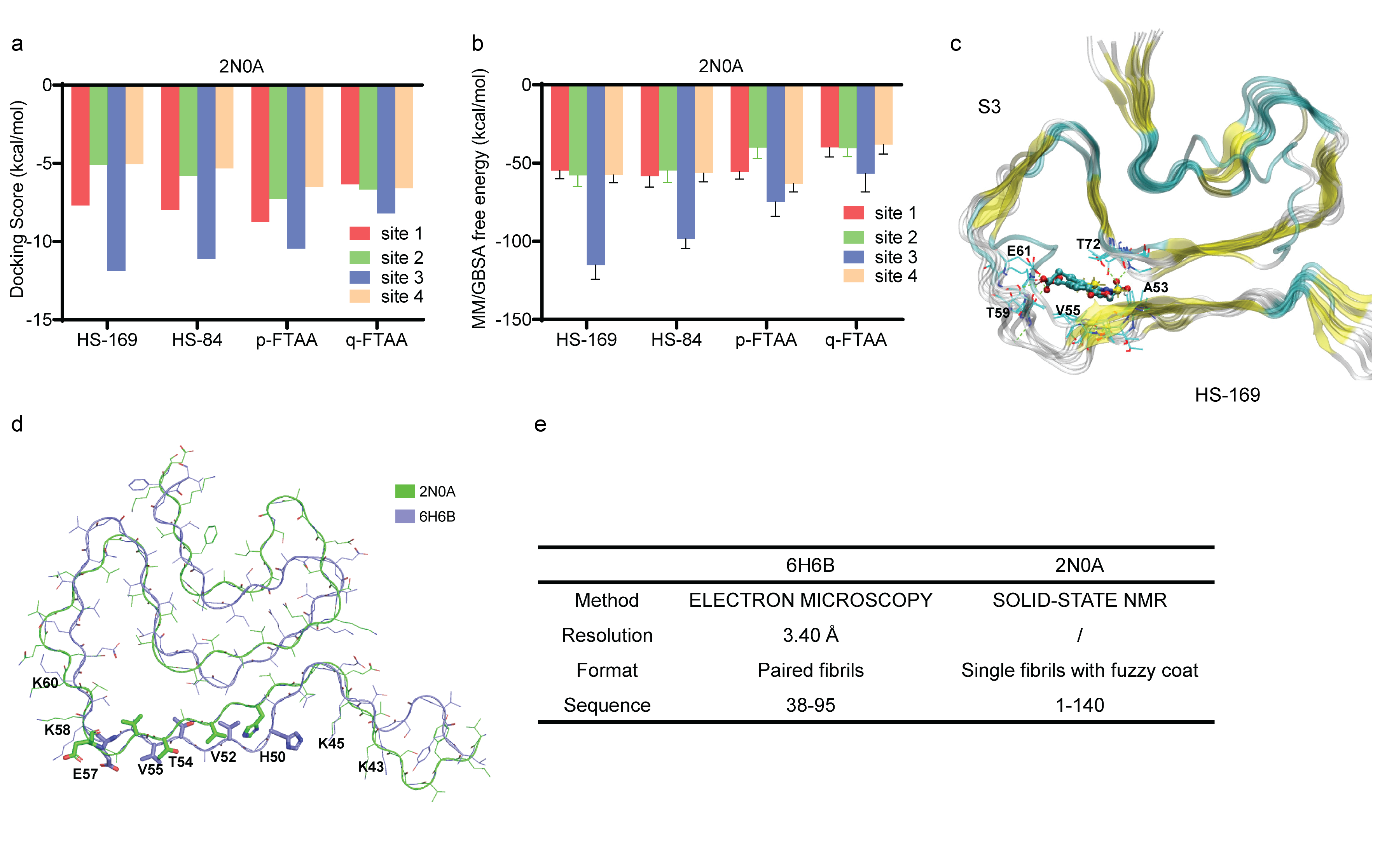
 **Supplementary Fig. 3**. **In silico modelling of the binding sites of HS-169, HS-84, h-FTAA, p-FTAA and q-FTAA on the 2N0A alpha-synuclein structure.** (**a, b**) Docking and MM/GBSA calculation of free energy indicating that site 3 is preferred by HS-169, HS-84, h-FTAA, p-FTAA and q-FTAA on the 2N0A alpha-synuclein structure. (**c**) Zoomed-in view of HS-169 binding to site 3. (**d, e**) Difference between the 2N0A and 6H6B structures.

**References**

1. Shirani H, Linares M, Sigurdson CJ, Lindgren M, Norman P, Nilsson KP. A Palette of Fluorescent Thiophene-Based Ligands for the Identification of Protein Aggregates. Chemistry. 2015;21:15133-7. doi:10.1002/chem.201502999.

2. Klingstedt T, Aslund A, Simon RA, Johansson LB, Mason JJ, Nystrom S, et al. Synthesis of a library of oligothiophenes and their utilization as fluorescent ligands for spectral assignment of protein aggregates. Organic & biomolecular chemistry. 2011;9:8356-70. doi:10.1039/c1ob05637a.

3. Burmann BM, Gerez JA, Matecko-Burmann I, Campioni S, Kumari P, Ghosh D, et al. Regulation of alpha-synuclein by chaperones in mammalian cells. Nature. 2019. doi:10.1038/s41586-019-1808-9.

4. Gerez JA, Prymaczok NC, Rockenstein E, Herrmann US, Schwarz P, Adame A, et al. A cullin-RING ubiquitin ligase targets exogenous alpha-synuclein and inhibits Lewy body-like pathology. Sci Transl Med. 2019;11. doi:10.1126/scitranslmed.aau6722.
